# Modulating Sound with Acoustic Metafiber Bundles

**DOI:** 10.1038/s41598-017-07232-6

**Published:** 2017-08-15

**Authors:** Jian-ping Xia, Hong-xiang Sun, Shou-qi Yuan

**Affiliations:** 1 Research Center of Fluid Machinery Engineering and Technology, Faculty of Science, Jiangsu University, Zhenjiang, 212013 China; 2 State Key Laboratory of Acoustics, Institute of Acoustics, Chinese Academy of Sciences, Beijing, 100190 China

## Abstract

Acoustic metamaterials and metasurfaces provide great flexibility for manipulating sound waves and promise unprecedented functionality, ranging from transformation acoustics, acoustic cloaking, acoustic imaging to acoustic rerouting. However, the design of artificial structures with both broad bandwidth and multifunctionality remains challenging with traditional design approaches. Here we present a design and realization of a broadband acoustic metafiber bundle. Very different from previously reported acoustic metamaterials and metasurfaces, not only the metafiber structure is simple, flexible and tunable, but also the metafiber bundle has the advantages of broad bandwidth, high transmission, no resonance-induced energy loss and unchangeable output wavefront owing to eigenmodes in the passbands of the metafiber. Besides, it could also achieve arbitrary complex modulations of cylindrical and plane acoustic wavefronts. The metafiber bundles realize the exciting multifunctionality of both acoustic metamaterials and metasurfaces in a broad frequency range, which provides diverse routes to design novel acoustic devices with versatile applications.

## Introduction

Recent years have witnessed the emergence of two types of wave modulation structures, known as metamaterials^1–9^ and metasurfaces^10–17^. These artificial structures of subwavelength sizes are capable of controlling the wave propagation in new ways, made possible by the creation of unusual material properties, which has attracted great attentions from both physics and engineering communities. Taking inspiration from developments in electromagnetism, acoustic metamaterials^18–36^ and metasurfaces^37–42^ have been developed rapidly in a wide range of potential applications, such as negative refraction of sound waves^21, 24, 27, 30, 31, 43^, ultrasonic imaging with subwavelength resolution^21–23, 44–46^, acoustic cloaking^47–55^, acoustic rectifiers^56–60^ and acoustic focusing, self-bending and vortexing beam generators^37–41, 61^.

So far, most acoustic metamaterials and metasurfaces have been denoted as a class of structured composites whose acoustic functionalities arise from the collective manifestations of their locally resonant elements. These designed resonant elements on a scale are much shorter than their operating wavelength, which provides their effective physical parameters with negative values. For example, the first metamaterial with negative dynamic mass density used rubber-coated spheres to realize locally resonant and deeply subwavelength structures that responded to incident acoustic waves^18^. Other locally resonant elements, such as Helmholtz resonators^19, 21, 41, 51^, tensioned membranes^20, 24, 29, 38, 58^, space-coiling structures^27, 30, 34, 37, 39, 40, 62^, hybrid elastic unit cells^25^ and porous structures^23, 42^, have also been successfully used to fabricate acoustic metamaterials and metasurfaces. Although these locally resonant elements have realized subwavelength sizes and diversiform novel functionalities, but they depend heavily on their resonant effects, and these functionalities decrease sharply when away from their resonant frequencies. Besides, most of these resonant elements usually have fabrication complexity, and are constructed only for some specific functionalities. The design of simple efficient elements with multifunctionality of both acoustic metamaterials and metasurfaces remains a challenge, which limits their practical applications.

In this work, we demonstrate a broadband acoustic metafiber bundle with the exciting multifunctionality of both acoustic metamaterials and metasurfaces. We demonstrate experimentally and numerically that, the ratio of operating bandwidth to center frequency could reach about 0.25, which is induced from the passband of the metafiber and is much broader than that of other resonant elements. Besides, owing to the eigenmodes in the passbands, the metafiber bundle has the advantages of high transmission, no resonance-induced energy loss and unchangeable output wavefront. Moreover, the metafiber bundle is simple, flexible and tunable. Based on these performances, we realize the invisibility of the plane and cylindrical acoustic sources, and design the acoustic shifter and acoustic beam splitter. We also demonstrate that the metafiber could steer a local phase shift that spans a full 2π range, which achieves arbitrary complex modulations of incident cylindrical and plane wavefronts in a broad frequency range. To verify this, we present the realization of the negative refractions with one or two beams and acoustic focusing by different types of metafiber bundles. The acoustic metafiber bundles with both broad bandwidth and multifunctionality are highly desirable in engineering applications, such as designs of acoustic cloaking, acoustic superlens, acoustic beam splitter and acoustic self-bending and vortexing beam generators.

## Results

### Broadband acoustic metafiber

**Figure 1:**
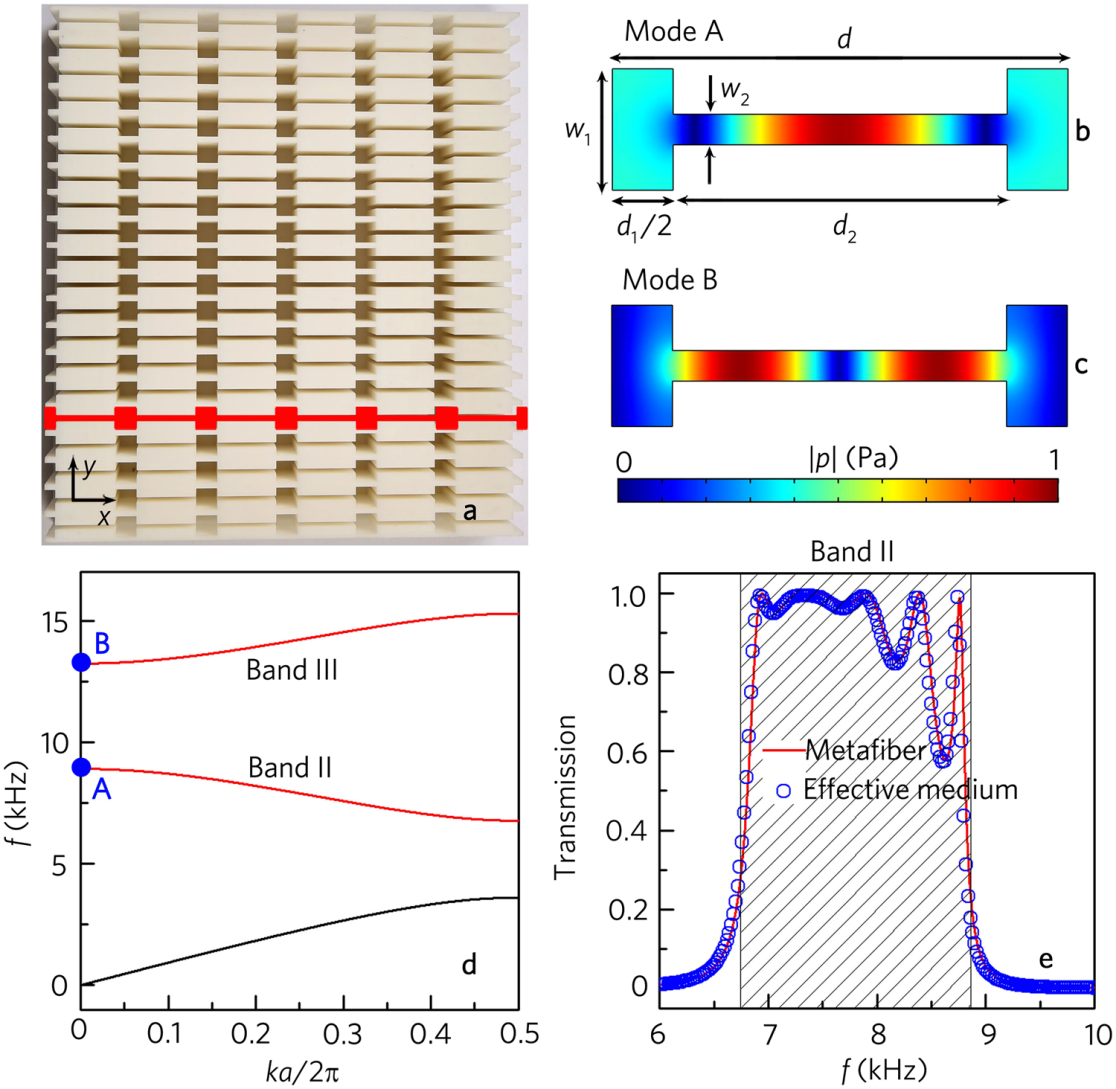
Geometry structure and mode analysis of acoustic metafiber. (**a**) Photograph of an acoustic metafiber bundle composed of twenty metafibers which is fabricated with photosensitive resins by means of 3D printing. The cover sheet is removed to view its structures, and the red illustration refers to a single acoustic metafiber. Simulated pressure eigenfunctions of a unit cell in the acoustic metafiber at *k* = 0. (**b**) Mode A, located at 8902 Hz. (**c**) Mode B, located at 13233 Hz. (**d**) Band structure of the acoustic metafiber, where the red lines represent bands II and III. Blue dots mark the eigenfrequencies shown in (**b**,**c)**. (**e**) Simulated transmission spectrum of the acoustic metafiber and that of the effective medium theoretically calculated by the transfer matrix method. Shaded region in **e** represent the band II of the acoustic metafiber.

As schematically shown in Fig. 1a, we adopt an acoustic metafiber bundle which consists of twenty metafibers to realize sound modulations, and the distance between two metafibers is 10mm. The one-dimensional acoustic metafiber marked in red is composed of six unit cells. Each unit cell with the lattice constant *d* has two wider rectangular cavities with the length 0.5*d*_1_ and width *w*_1_ = 8 mm_,_ sandwiching a narrow rectangular cavity with the length *d*_2_ and width *w*_2_ = 2 mm. The cavities are filled with air and made of photosensitive resins to meet the sound hard boundary condition. An example of a unit cell, with *d*_1_ = 8 mm and *d*_2_ = 22 mm, is shown in Fig. 1b,c, together with simulated pressure eigenfunctions of the lower two eigenmodes (Mode A and Mode B) at *k* = 0. Their corresponding eigenfrequencies are marked by two blue dots in Fig. 1d, which shows the band structure of the metafiber. The two eigenmodes which are the band states of the bands II and III (shown by red curves in Fig. 1d) have similar pressure gradient along the *x* direction and negligible pressure variation along the *y* direction. Throughout this work, we study the performances of the metafiber structure in the band II (Fig. 1b) in detail.

Figure 1e presents the transmission spectra of the metafiber, in which the shadow region represents the band II. It is worth pointing out that the operating bandwidth with high transmission reaches about 2.0 kHz, and the ratio of the bandwidth to the center frequency is up to 0.25, which is attributed to the eigenmode (Fig. 1b) of the metafiber and is much broader than that of other resonant elements^27, 41^. Besides, the metafiber has high transmission efficiency in the operating band, and the transmission is close to 1.0 from 7.0 kHz to 8.0 kHz. The simulated transmission of the metafiber agrees well with that of the effective medium theoretically calculated by the transfer matrix method (Supplementary Note 1).

**Figure 2:**
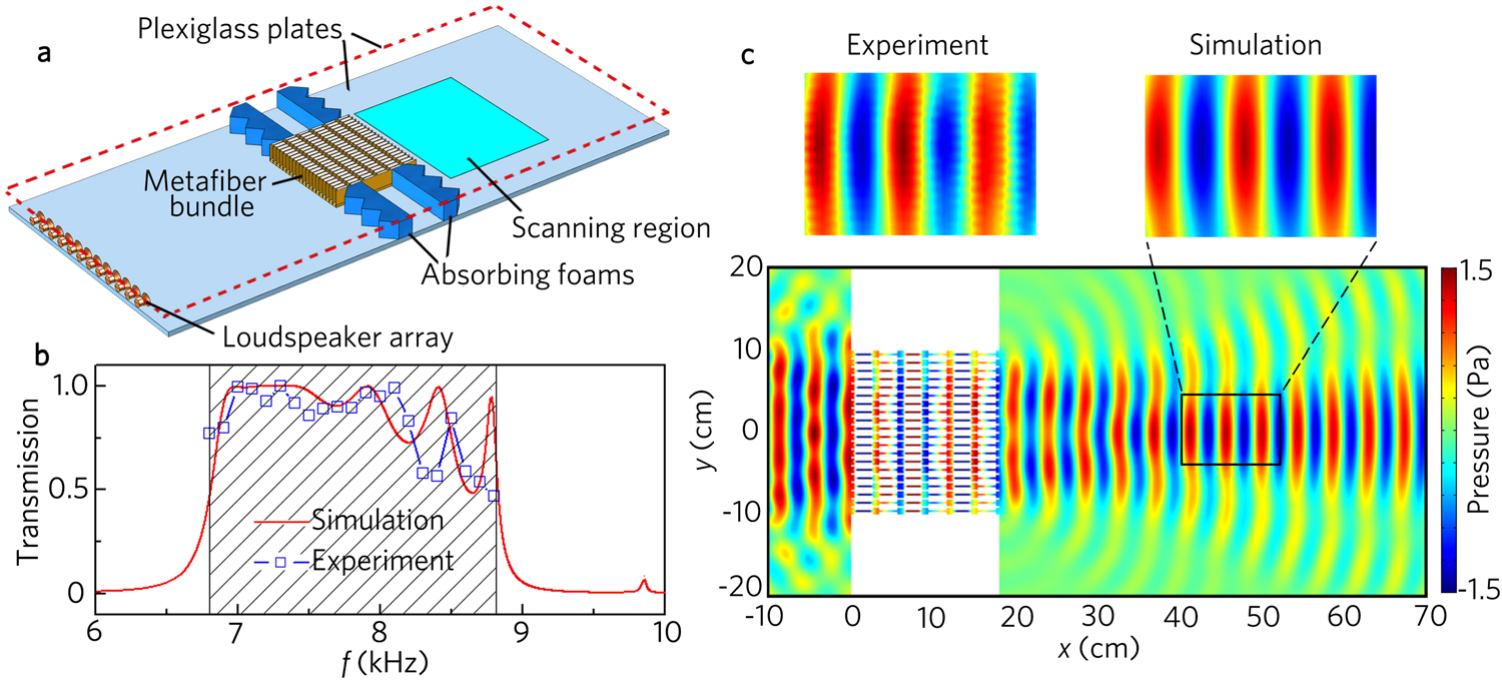
Transmission performance. (**a**) Schematic of the experimental set-up. The top plate of the waveguide (not shown in the diagram) is located 10 mm above the bottom plate. (**b**) Simulated and measured transmission spectra with the acoustic metafiber bundle shown in **1a**. (**c**) Simulated and measured acoustic pressure field distributions with the acoustic metafiber bundle illuminated by the normally incident plane wave at 8.0 kHz.

To verify these performances, we have experimentally measured its transmission properties. Figure 2a shows the schematic diagram of the experimental setup, in which the metafiber bundle is shown in Fig. 1a. As shown in Fig. 2b, the transmittance spectra are agreeable very well between the experimental measurements and numerical simulations. Figure 2c shows the comparison between the simulated and the measured pressure fields of transmitted wave with the metafiber bundle. It is clear that the plane wave entirely transmits through the metafiber, and the transmitted wavefront is the same as the incident wavefront. In addition, the measured transmitted wave is still a perfect plane wave, and the experimental results agree well with the numerical ones. The results indicate that the metafiber has the performances of high transmission, no resonance-induced energy loss and unchangeable output wavefront.

### Performance of acoustic invisibility

**Figure 3:**
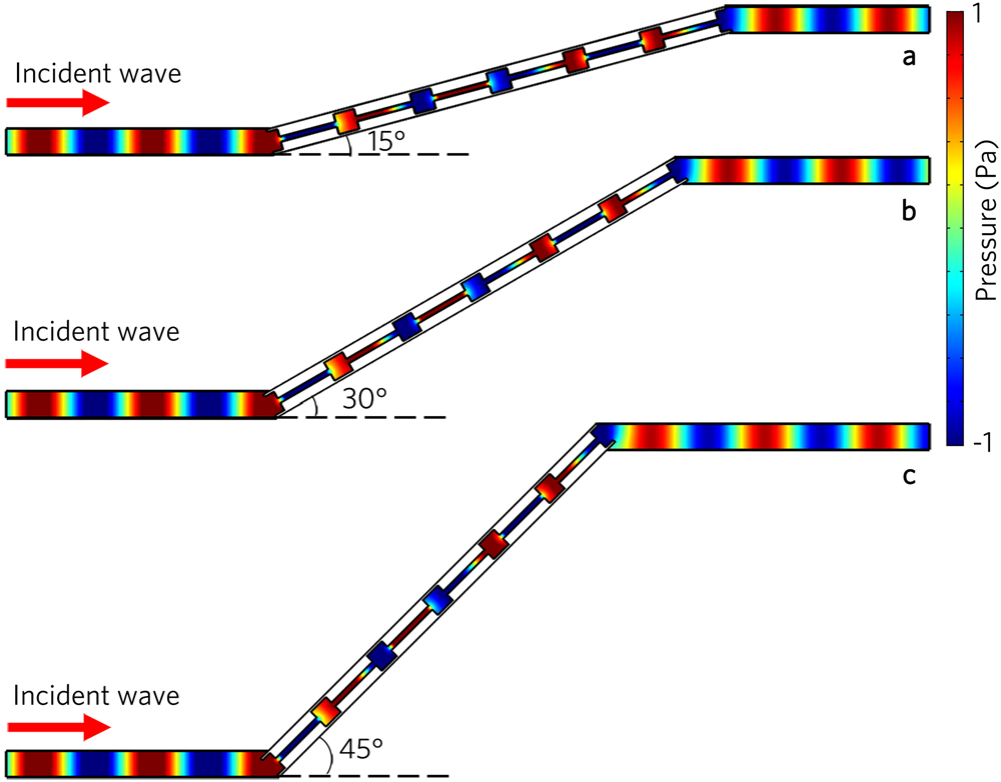
Acoustic propagation path modulations. Simulated acoustic pressure field distributions for the acoustic metafibers with different rotation angles (**a**) 15°, (**b**) 30° and (**c**) 45°, illuminated by the normally incident plane wave at 8.0 kHz.

The acoustic metafiber is simple, flexible and tunable, which is capable of controlling the acoustic propagation path by rotating the metafibers. As an example, we rotate the metafiber 15°, 30° and 45°, respectively. As shown in Fig. 3a–c, the acoustic waves also entirely pass through the metafibers with different rotation angles. Besides, with the increase of the rotation angle, the transmitted wavefronts remain unchanged, but the propagation paths move upward gradually, which provides the feasibility of the acoustic invisibility.

**Figure 4:**
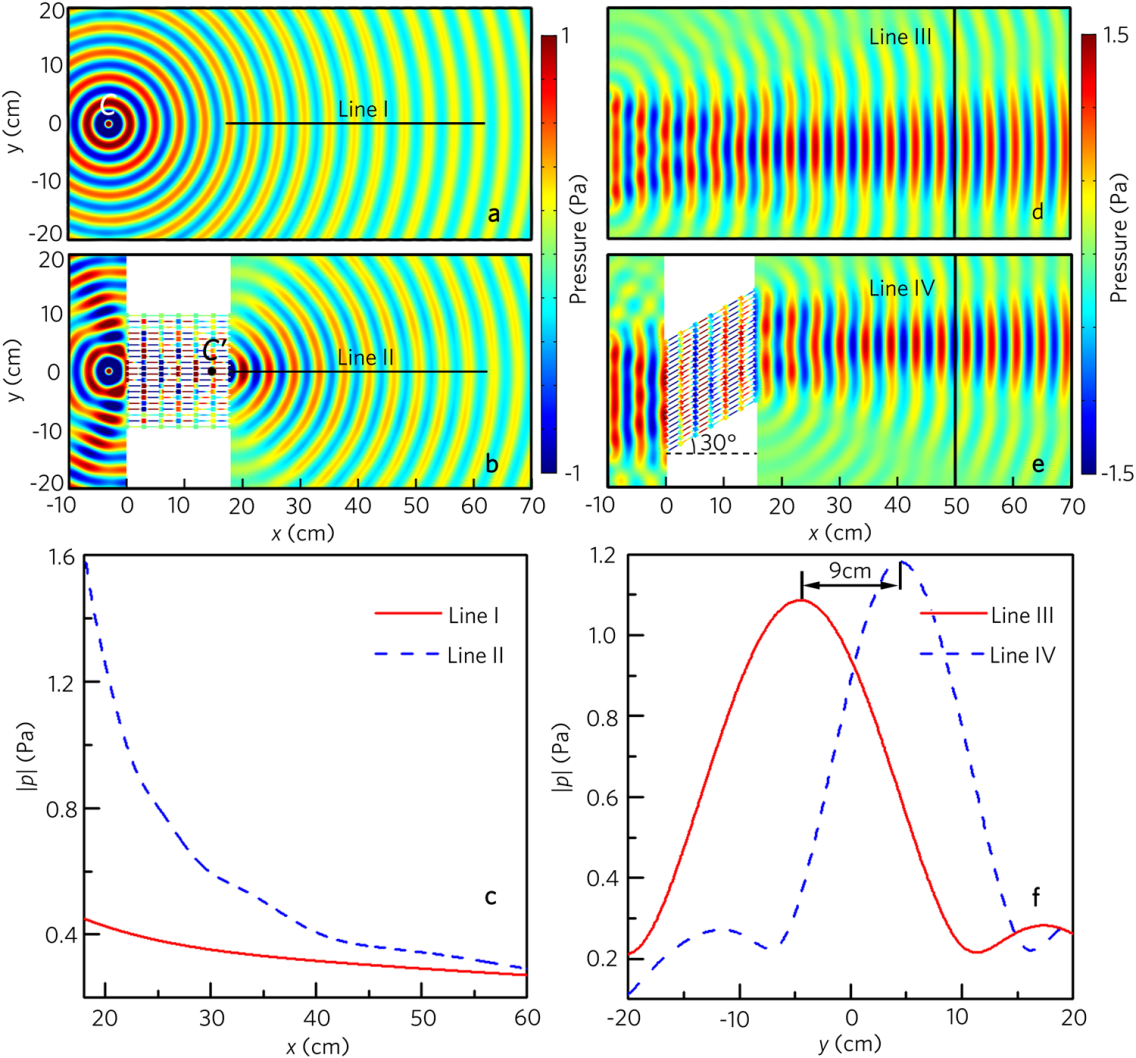
Acoustic invisibility performances. Simulated acoustic pressure field distributions (**a**) without and (**b**) with the acoustic metafiber bundle illuminated by an incident cylindrical wave at 8.0 kHz. The cylindrical acoustic source is located at (−3 cm, 0) in Cartesian coordinates. (**c**) Pressure amplitude distributions along the lines I and II in (**a** and **b**). Simulated acoustic pressure field distributions (**d**) without and (**e**) with the acoustic metafiber bundle illuminated by the normally plane wave at 8.0 kHz, and the acoustic metafiber bundle in **e** is rotated 30°. (**f**) Pressure amplitude distributions along the lines III and IV in (**d** and **e**).

To demonstrate this performance, we simulate the distribution of the pressure field induced by a cylindrical wave without and with the metafiber bundle (the same as that in Fig. 1a), which is shown in Fig. 4a,b. In the free space, the cylindrical wave field is undisturbed, and is centered on the incident point *C* [Fig. 4a]. As shown in Fig. 4b, the cylindrical wave can transmit through the metafiber bundle, and the transmitted wavefront is unchanged. It is interesting to note that to the observer in the right, the cylindrical wave appears to be excited at the point which is a perfect virtual image of *C*. Figure 4c presents the distributions of the pressure amplitude along the lines I [Fig. 4a] and II [Fig. 4b]. The pressure amplitude induced by the cylindrical wave decreases rapidly in the free space, but the cylindrical wave does not attenuate passing through the metafiber bundle. It looks like that the transmitted pressure field is produced by the cylindrical acoustic source at the point, and the original acoustic source is hidden by the metafiber bundle.

In the case of the plane acoustic source, we adopt the metafiber bundle with the rotation angle of 30°, and other parameters are the same as those in Fig. 1a. As shown in Fig. 4d,e, the plane wave field is undisturbed in the free space. With the metafiber bundle, the transmitted wavefront keeps unchanged, but the propagation path moves upward greatly. Figure 4f shows the distributions of the pressure amplitude along the lines III [Fig. 4d] and IV [Fig. 4e]. Note that the distance between both pressure peaks is about 9 cm, which indicates that, with the metafiber bundle, the incident position appears to be upper than its real position.

**Figure 5:**
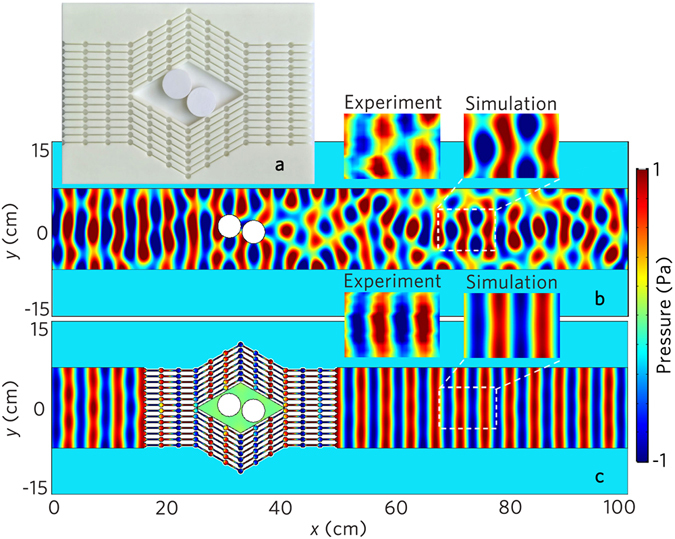
Acoustic shifter. (**a**) Photograph of the designed acoustic shifter. Simulation results of the acoustic pressure field distributions (**b**) without and (**c**) with the acoustic shifter illuminated by the normally incident plane wave at 8.0 kHz. Two white circular objects with the radius of 2 cm in (**a**–**c**) represent the rigid scatterers.

Owing to the aforementioned advantages and the flexible structure of the acoustic metafiber bundles, we design an acoustic shifter by placing metafiber bundles in sound propagation paths. The photograph of an acoustic shifter is shown in Fig. 5a, in which the unit cell of the metafiber consists of two circular cavities with the radius of 4mm and a rectangular cavity for easy connection. As shown in Fig. 5b, two rigid circular scatterers are placed at random positions in a waveguide with the height of 14 cm, and the incident plane wave is disturbed seriously by both scatterers. However, with the acoustic shifter (Fig. 5c), the acoustic waves propagate along the designed propagation pathes and round the scatterers. The wavefront and amplitude of the transmitted waves are unchanged, and the scatterers are hidden by the acoustic shifter. Interesting application of this property in the design of the acoustic beam splitter is further discussed (Supplementary Note 2).

### Acoustic wavefront modulations

**Figure 6:**
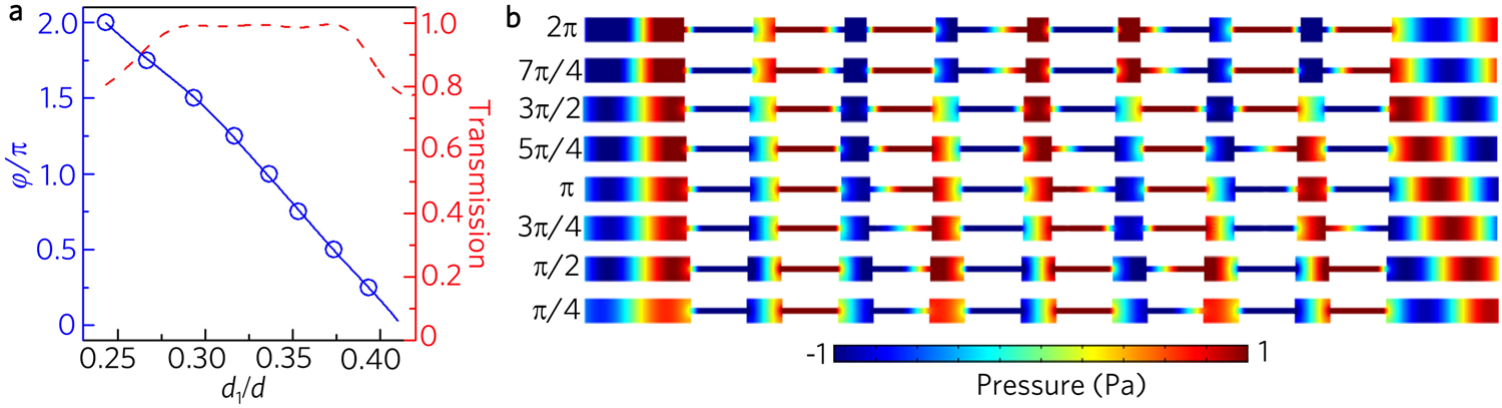
Acoustic wavefront modulations. (**a**) Phase shift (blue solid line) and transmission spectrum (red dashed line) of the acoustic metafiber as a function of the tunable cavity length ratio (*d*_1_/*d*). (**b**) Simulated acoustic pressure field distributions through eight different acoustic metafibers for an equally increased phase shift with a step of π/4, corresponding to eight hollow blue dots *d*_1_/*d* = 0.243, 0.267, 0.293, 0.317, 0.337, 0.353, 0.373 and 0.393 in (**a**).

The acoustic metafiber could also be used to modulate acoustic wavefronts, which is similar to the performances of metasurfaces. To show this performance, we choose an acoustic metafiber with eight unit cells. Figure 6a shows the transmission spectrum and phase shift *φ* of the metafiber as a function of the tunable cavity length ratio (*d*_1_/*d*), and the lattice constant *d* = 30 mm keeps unchanged. In the range 0.24 ≤ *d*_1_/*d* ≤ 0.42, the transmission is larger than 0.8, and the phase shift could cover the whole 2π range. To verify the phase shifts of the metafiber, we select eight values of *d*_1_/*d* [shown by the eight blue hollow dots in Fig. 6a] to realize eight steps of an equally spaced phase shift from 0 to 2π. The pressure fields generated by the eight different metafibers are shown in Fig. 6b, where the acoustic wave is normally incident from the left side. The simulated results clearly show the desired phase shift equally spaced from 0 to 2π with a step of π/4.

**Figure 7:**
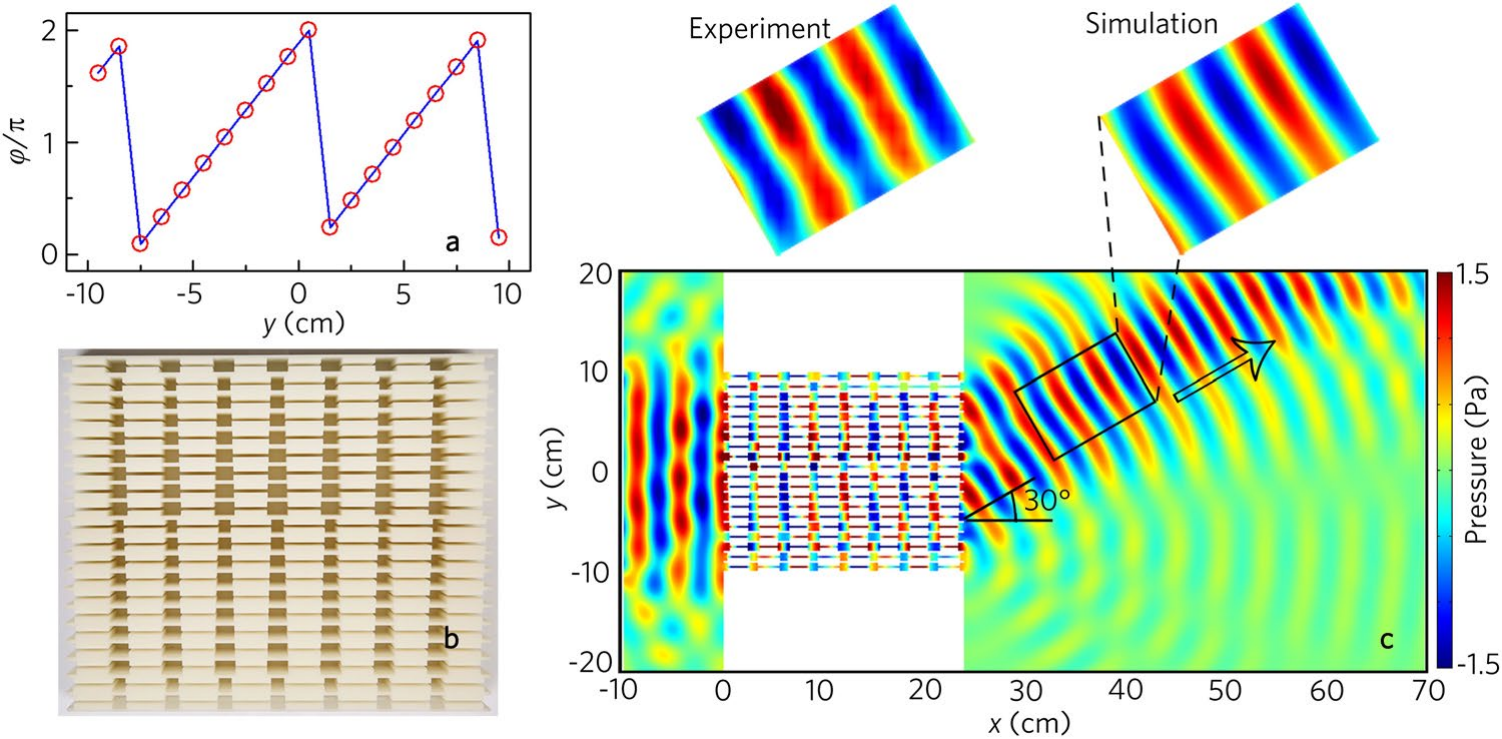
Acoustic negative refraction with a beam. (**a**) Distribution of twenty acoustic metafibers along *y* direction as indicated by their discrete phase shift (twenty red hollow dots) resembling the desired phase profile *φ*(*y*) (blue line). (**b**) Photograph of the designed metafiber bundle. (**c**) Simulated and measured acoustic pressure field distributions with the acoustic metafiber bundle illuminated by the normally incident plane wave at 8.0 kHz. Black arrow in (**c**) refers to the theoretical value of the refraction.

The realization of 2π range phase shifts of transmitted waves allows us to revisit the Snell’s law by choosing appropriate phase profiles. Considering the acoustic waves with normal incidence along the *x* direction, due to the existing of discrete phase shifts along the *y* direction, the refraction angle of the transmitted wave *θ*_*t*_ (measured from the *x* direction) can be deduced by applying the generalized Snell’s law^10^

1 $${\theta }_{t}={\rm{arc}}\,\sin (\frac{1}{k}\frac{{\rm{d}}\phi (y)}{{\rm{d}}y}),$$

where *φ*(*y*) and d*y* are the phase shift and the distance between two metafibers along the *y* direction respectively, and *k* = 2π/*λ* is the wave vector in air. According to Eq. 1, we can realize arbitrary refraction angles by designing the appropriate phase profile along the *y* direction. To demonstrate this feature, we choose the refraction angle *θ*_*t*_ = π/6. The distribution of twenty metafibers along the *y* direction is illustrated by the discrete phase shifts shown by red hollow dots in Fig. 7a, and the discrete phase shifts are determined by the desired continuous phase profile [cf. blue curve in Fig. 7a].

Figure 7b shows the photograph of the designed metafiber bundle, and the simulated and the measured results of the transmitted pressure fields with the metafiber bundle are displayed in Fig. 7c. The experimental results agree well with the numerical ones, showing the existence of negative refraction along the designed direction (viz., 30°). Besides, we simulate the wavefront modulations with the same metafiber bundle in the range from 7.4 kHz to 8.8 kHz (Supplementary Note 3), which demonstrates that the acoustic metafiber bundles can be applied to the wavefront modulations in a broad frequency range.

**Figure 8:**
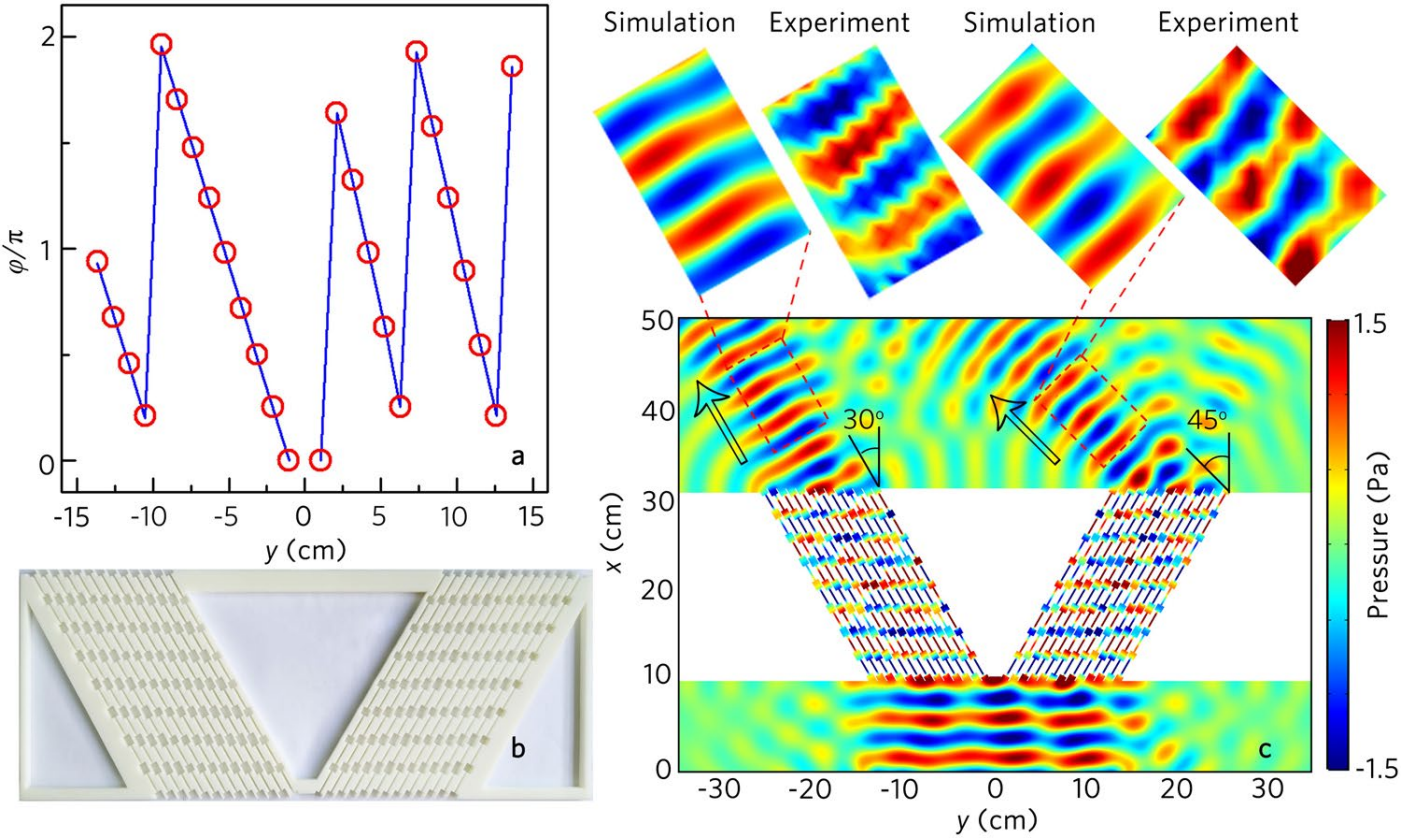
Acoustic negative refraction with two beam. (**a**) Distribution of twenty-six acoustic metafibers along *y* direction as indicated by their discrete phase shift (twenty-six red hollow dots) resembling the desired phase profile *φ*(*y*) (blue line). (**b**) Photograph of the designed metafiber bundle. (**c**) Simulated and measured acoustic pressure field distributions with the acoustic metafiber bundle illuminated by the normally incident plane wave at 8.0 kHz. Black arrow in (**c**) refers to the theoretical value of the refraction.

We also realize the negative refraction with two acoustic beams by using the acoustic metafiber. In this case, we introduce two metafiber bundles, and each consists of thirteen metafibers. The distance between two metafibers is 10.5 mm. To make full use of the incident acoustic energy, we rotate both the metafiber bundles 30° along the opposite direction. As an example, we choose two different refraction angles *θ*_*t*1_ = 30° and *θ*_*t*2_ = 45°. Figure 8a shows the phase shift of both metafiber bundles, in which the blue lines refer to the desired theoretical phase profile, and the red hollow dots represent the discrete phase profile of the twenty-six metafibers.

Figure 8b shows the photograph of the designed metafiber bundle, and the simulated and the measured results of the transmitted pressure fields are displayed in Fig. 8c. As shown in Fig. 8c, two transmitted acoustic beams with the refraction angles 30° and 45° are formed by the modulations of the metafiber bundles, and both transmitted beams are almost undistorted. Besides, the transmitted pressure fields are agreeable very well between the experimental measurements and the numerical simulations.

**Figure 9:**
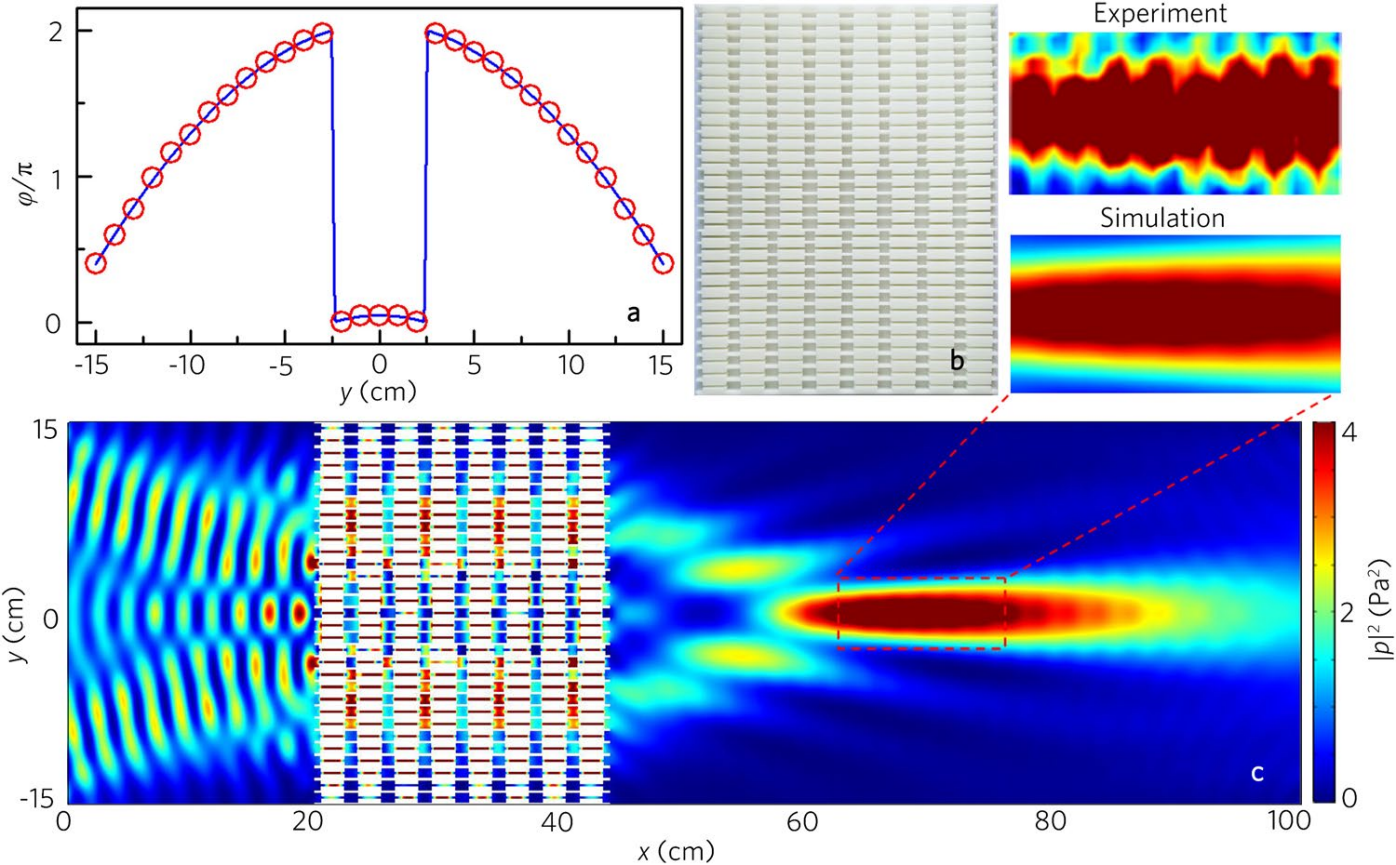
Acoustic focusing. (**a**) Distribution of thirty-one acoustic metafibers along *y* direction as indicated by their discrete phase shift (thirty-one red hollow dots) resembling the desired phase profile *φ*(*y*) (blue line). (**b**) Photograph of the designed focusing lens. (**c**) Simulated and measured acoustic pressure field distributions with the acoustic focusing lens illuminated by the normally incident plane wave at 8.0 kHz.

In addition to the aforementioned results, we present the realization of the focusing lens by using the metafiber bundle. For a given focal length *f*, the phase shift must satisfy the following equation $\phi {\boldsymbol{(}}y)=-k\sqrt{{y}^{2}+{f}^{2}}$. By applying this equation, the desirable continuous phase profile (blue line) and the discrete phase profile (red hollow dots) provided by the metafiber are plotted in Fig. 9a. The focusing lens is fabricated based on the discrete phase profile, and the photograph of the designed metafiber bundle is shown in Fig. 9b. Spatial intensity distributions of the focusing lens with *f* = 30 cm is shown in Fig. 9c. It is found that the transmitted waves are focused in the experimental and numerical results, and the experimental acoustic intensity fields agree with the numerical ones. This indicates that the metafiber bundle could realize arbitrary complex modulations of the plane acoustic sources. Besides, we further discuss the complex wavefront modulations of the cylindrical acoustic sources, such as negative refractions with one or two beams and focusing (Supplementary Note 4).

## Discussion

We have proposed and demonstrated a broadband acoustic metafiber bundle, which has the exciting multifunctionality of both acoustic metamaterials and metasurfaces. We demonstrate experimentally and numerically that, the ratio of operating bandwidth to center frequency is up to 0.25, which is much broader than that of other resonant elements. Besides, owing to the eigenmodes in the passbands of the metafiber, the metafiber bundle has the advantages of high transmission, no resonance-induced energy loss and unchangeable output wavefront, and its structure is simple, flexible and tunable, which is very desirable in many useful applications such as acoustic cloaking, acoustic super-resolution imaging and acoustic beam splitter and so on. On the basis of these performances, we demonstrate the invisibility of the plane and cylindrical acoustic sources, and design the acoustic shifter and acoustic beam splitter. The metafiber could also steer a local phase shift that spans a full 2π range, which achieves arbitrary complex modulations of incident cylindrical and plane wavefronts in a broad frequency range, and can be applied to underwater sound arrays, audio presentation, particle manipulation and ultrasound imaging and therapy. To verify this, we present the negative refractions with one or two beams and acoustic focusing by different types of metafiber bundles. Our work provides a fertile ground for sound modulations with broad bandwidth. With the acoustic metafiber bundles, we can expect novel acoustic cloaking, rerouting and imaging applications in the near future.

## Methods

### Sample fabrication and experimental setup

The designed acoustic metafiber bundles are made of photosensitive resin, and are manufactured via three-dimensional printing technology. In the planar waveguide system, we have adopted two paralleled plates (dimension 1 m × 4 m × 1 cm). Wedge-shaped sound absorbing foams are installed at the boundaries of the planar waveguide and the two sides of the sample to mimic an anechoic environment. To generate the incident plane waves required for the experiment, nine loudspeakers (2.8 cm × 4.0 cm) are assembled into a liner array with an interval of 4.5 cm. All the speakers are synchronously driven by a power amplifier which is controlled by the controller model (Brüel & Kjær 3160-A-022) with the software PULSE Labshop.

Two 0.25-inch-diameter microphones (Brüel&Kjær type-4961) are used to measure the acoustic pressure. One microphone is put into the measurement area through a square hole (20 cm × 20 cm) in the upper plate to detect the pressure as signal 1, and it can be moved flexibly by a set of two-dimensional motorized linear stages (Newport: MIN300CC and ILS250CC) controlled by the model (Newport: ESP301-3N). The other is fixed towards the sample to detect the pressure as signal 2. In the pressure mapping measurement, we obtain the phase assigned spectrum of the two signals detected by the two microphones with signal 1 works as an input signal and signal 2 as a reference. By using the software PULSE Labshop, we could retrieve the pressure field by recording the pressure magnitude and phase of each position in the scanning area. In the transmission spectra measurement, the distance between the detecting microphone and the metafiber bundle is 30 cm, and the transmission could be obtained by recording the pressure magnitudes with and without metafiber bundles, respectively.

### Numerical simulations

The finite element method (COMSOL Multiphysics software 5.2a) is utilized to numerically simulate the characteristics of the acoustic metafiber bundles. The boundaries in the metafiber bundle are set as sound hard boundary. The pressure field is calculated in the Acoustic-Solid interaction module, and the boundaries of the simulated area are set as plane wave radiation boundary. The materials are air and photosensitive resin. The parameters used for air under an ambient pressure of 1 atm at 20 °C are mass density *ρ*_air_ = 1.21 kg/m^3^ and sound speed *c*_air_ = 344 m/s. The mass density, Young’s modulus and Poisson’s ratio for photosensitive resin are 1050 kg/m^3^, 5.08 GPa and 0.35, respectively.

## Electronic supplementary material

Supplementary information
