# Supplementary material for: Modulating Sound with Acoustic Metafiber Bundles
Source: Sci Rep. 2017 Aug 15;7:8151. doi: 10.1038/s41598-017-07232-6 (PMC5557887; doi:10.1038/s41598-017-07232-6)
Supplement: Supplementary file 1 — Supplementary information [file 41598_2017_7232_MOESM1_ESM.doc]

Supplementary Materials For

**Modulating Sound with Acoustic Metafiber Bundles**

Jian-ping Xia^1^, Hong-xiang Sun[[1]](#footnote-2), 2, Shou-qi Yuan^1^

**Supplementary Note 1**

**Effective medium model**

We develop an effective medium (EM) model to theoretically calculate the transmission spectrum based on the transfer matrix method. As shown in Supplementary Fig. 1a, the narrow rectangular cavity of the metafiber can be equivalent to an EM model which is shown in the blue region, and the width of the EM region is the same as that of the wider rectangular cavities. Each effective unit cell with the lattice constant *d* has two rectangular air regions with the length 0.5*d*_1_, sandwiching a rectangular EM region with the length *d*_2_. The transfer matrix$T_{0}$of an effective unit cell is expressed as^[1]^

$T_{0}=\left[\begin{matrix} e^{ik_{\text{air}}\frac{d_{1}}{2}} & 0 \\ 0 & e^{-ik_{\text{air}}\frac{d_{1}}{2}} \end{matrix}\right]\frac{1}{2}\left[\begin{matrix} \frac{Z_{air}+Z_{EM}}{Z_{EM}} & \frac{Z_{EM}-Z_{air}}{Z_{EM}} \\ \frac{Z_{EM}-Z_{air}}{Z_{EM}} & \frac{Z_{air}+Z_{EM}}{Z_{EM}} \end{matrix}\right]\left[\begin{matrix} e^{ik_{EM}d_{2}} & 0 \\ 0 & e^{-ik_{EM}d_{2}} \end{matrix}\right]\frac{1}{2}\left[\begin{matrix} \frac{Z_{EM}+Z_{air}}{Z_{air}} & \frac{Z_{air}-Z_{EM}}{Z_{air}} \\ \frac{Z_{air}-Z_{EM}}{Z_{air}} & \frac{Z_{EM}+Z_{air}}{Z_{air}} \end{matrix}\right]\left[\begin{matrix} e^{ik_{\text{air}}\frac{d_{1}}{2}} & 0 \\ 0 & e^{-ik_{\text{air}}\frac{d_{1}}{2}} \end{matrix}\right]$ (1)

where *k*_air_ and *k*_EM_ represent the wave number in air and EM, respectively, and *Z*_air_ and *Z*_EM_ represent the acoustic impedance of air and EM, respectively. The acoustic wave number *k*_EM_ and the acoustic impedance *Z*_EM_ are determined by the effective density *ρ*_eff_ and the effective sound speed *v*_eff_. As shown in Supplementary Fig. 1b, the metafiber with six unit cells could be treated as an effective one-dimensional periodic structure in the position from *x*_0_ to *x*_1_, in which the periodic structure is also composed of six effective unit cells. Thus, the transfer matrix$T_{m}$of the periodic structure can be expressed as$T_{m}=T_{0}^{6}$.


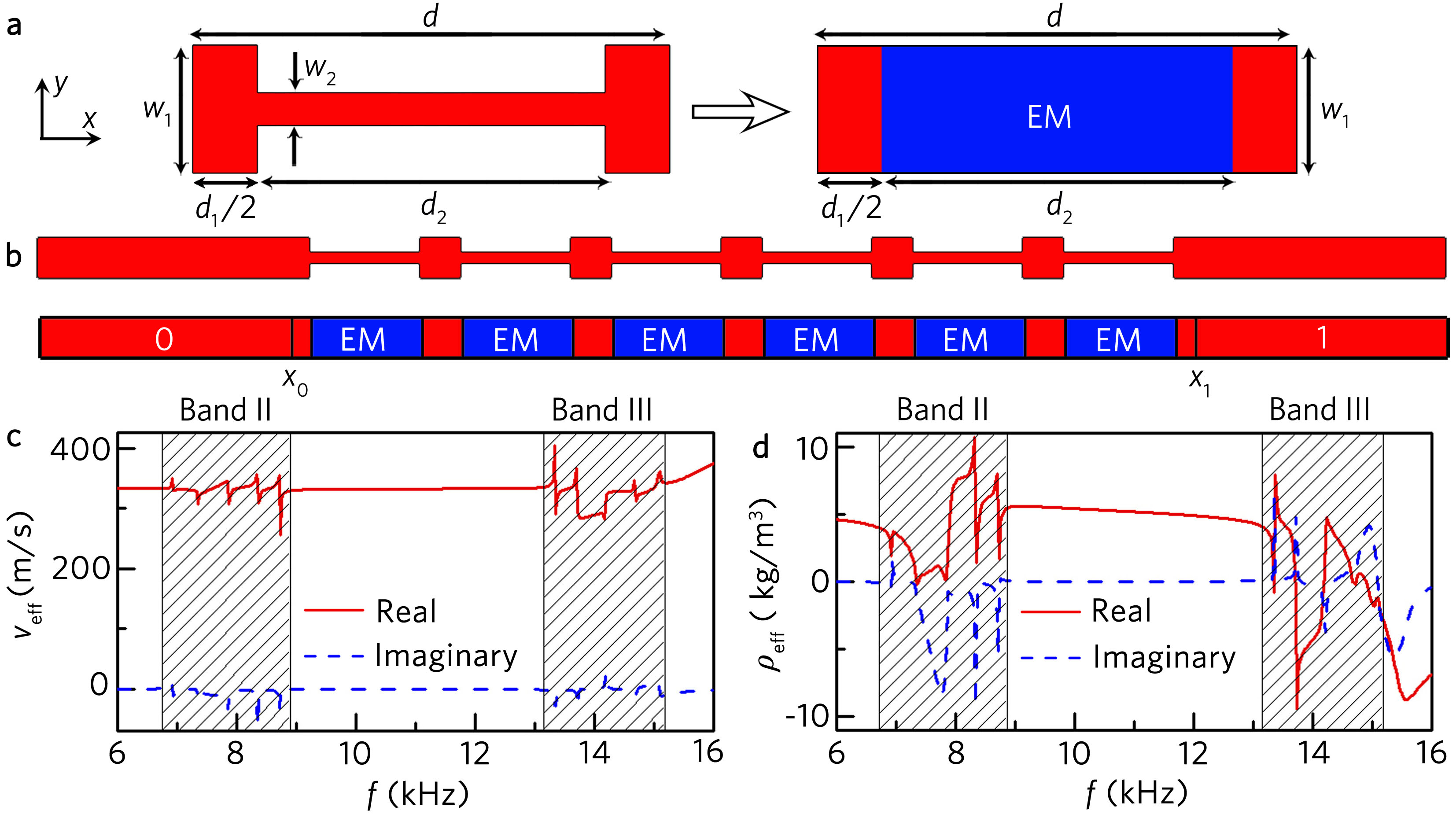


**Supplementary Figure 1** | **EM model**. (**a**) Schematic of a unit cell of the metafiber and its EM model, in which the blue and red regions refer to the EM and air. (**b**) Schematic of the metafiber with six unit cells and its EM model. (**c**) Effective sound speed and (**d**) density of the EM.

The acoustic pressure can be expressed as

$P_{n}=(p_{n}^{+}e^{ikx}+p_{n}^{-}e^{-ikx})e^{-i\omega t}$, (2)

where the subscript *n*=0 and 1 represents the left and right regions of the periodic structure. The$p_{n}^{+}$and$p_{n}^{-}$are the pressure amplitudes of the acoustic propagations along the positive and negative directions of *x*-axis, respectively. In the frequency domain, the pressure$P_{n}$can be rewritten as the matrix form­

$P_{n}=\left[\begin{matrix} p_{n}^{+}e^{ikx} \\ p_{n}^{-}e^{-ikx} \end{matrix}\right]$. (3)

We assume that the acoustic wave incident from the left side, thus, the acoustic pressures in the regions 0 and 1 are expressed as follows

$P_{0}=\left[\begin{matrix} p_{0}^{+}e^{ikx} \\ p_{0}^{-}e^{-ikx} \end{matrix}\right]$, (4)

$P_{1}=\left[\begin{matrix} p_{1}^{+}e^{ikx} \\ 0 \end{matrix}\right]$, (5)

where the$p_{0}^{+}$is the amplitude of the incident acoustic waves. Using the transfer matrix$T_{m}$, the relationship between$P_{0}$and$P_{1}$is

$P_{0}=T_{m}P_{1}$. (6)

Here, we define the complex transmission (*T*) and reflection (*R*) cofficients as

$T=p_{1}^{+}e^{ikx_{1}}/p_{0}^{+}e^{ikx_{0}}$, (7)

$R=p_{0}^{-}e^{ikx_{0}}/p_{0}^{+}e^{ikx_{0}}$. (8)

Based on Eqs. 6-8, we obtain

$\left[\begin{matrix} 1 \\ R \end{matrix}\right]=T_{\text{m}}\left[\begin{matrix} T \\ 0 \end{matrix}\right]$. (9)

Next, we calculate the transmission *T* and reflection *R* of the metafiber with six unit cells by using COMSOL Multiphysics software. Besides, *T*_m_ is a matrix which only contains both effective parameters *ρ*_eff_ and *v*_eff_. Therefore, we could retrieve both effective parameters *ρ*_eff_ and *v*_eff_ by solving Eq.9^[2]^ which are shown in Supplementary Fig. 1c-d. It is found that the effective parameters change obviously in bands II and III owing to the enginmodes of the metafiber.

Supplementary Figure 2a-b shows the distributions of the pressure field induced from the metafiber with six unit cells and its EM model. We find that the distributions of the pressure field are almost the same in Supplementary Fig. 2a-b. Supplementary Figure 2c presents the transmission spectra of the metafiber with six unit cell and its EM model in band III, in which the transmission spectra also agree well in this frequency range. Moreover, the effective parameters *ρ*_eff_ and *v*_eff_ obtained from the transmission and reflection of the metafiber with six unit cells could be used to calculate the acoustic properties of the metafiber with different number of unit cells. To verify this, we calculate the transmission spectra of the metafiber with ten unit cells and its EM model in bands II and III, which is shown in Supplementary Fig. 2d. Note that the transmission spectra also remain the same. It is indicated that the proposed EM model has good applicability.


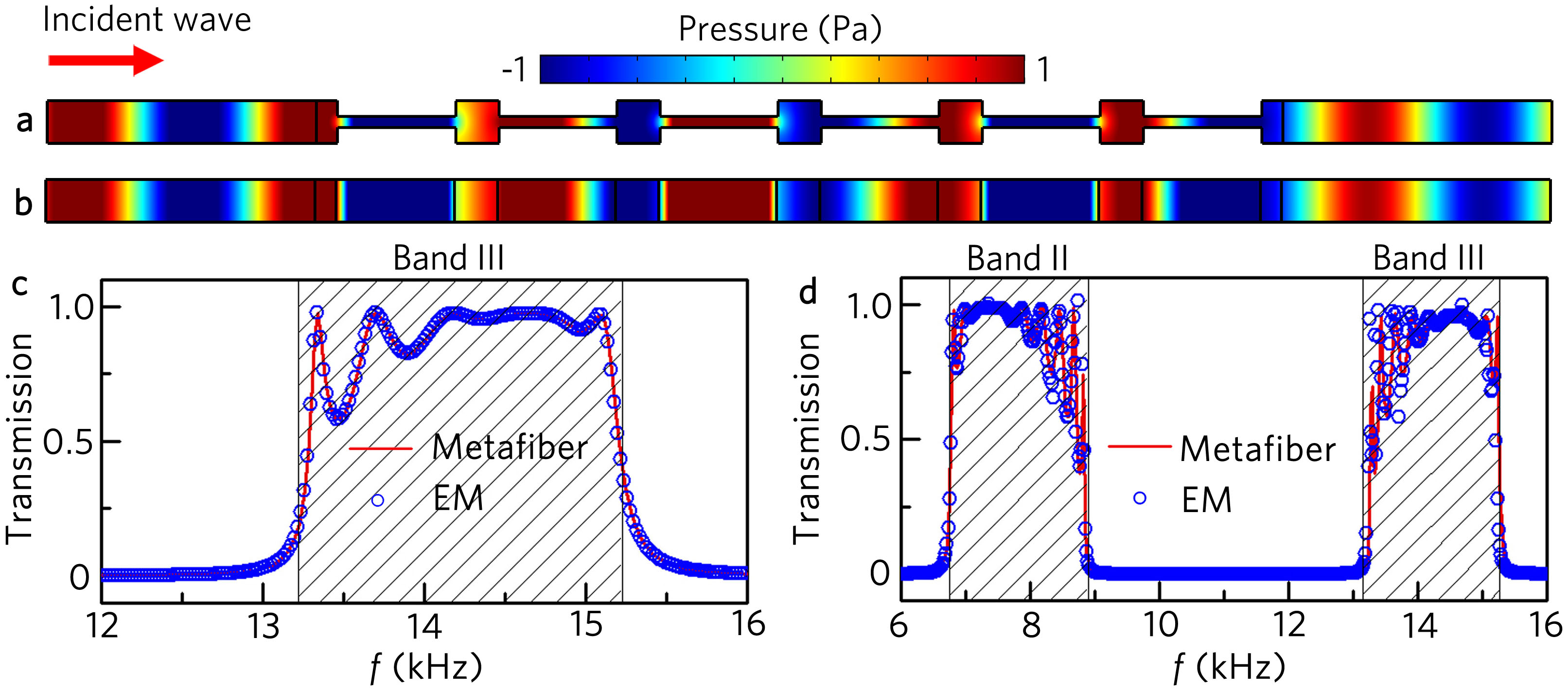


**Supplementary Figure 2 | Performances of EM model.** Simulation results of the pressure field distributions with **(a)** the acoustic metafiber with six unit cells and (**b**) its EM model illuminated by a normally incident plane wave at 8.0kHz, and the effective parameters in **b** are *ρ*_eff_=7.33-0.97i kg/m^3^ and *v*_eff_=332.65-2.32i m/s. Simulated transmission spectra of the acoustic metafibers with (**c**) six and (**d**) ten unit cells and those of their EM models theoretically calculated by the transfer matrix method.

**Supplementary Note 2**

**Acoustic beam** **splitter**

The proposed metafiber bundle could be used to design the acoustic beam splitter. As shown in Supplementary Fig. 3, the acoustic beam splitter consists of two metafiber bundles, and each has twenty uniform metafibers. The distance between two metafibers is 10.5mm, and both the metafiber bundles are rotated 30° along the opposite direction. It is clearly that the incident plane wave could transmit through the metafiber bundles and divide into two beams, in which both transmitted beams are undistorted, and the output and input wavefronts are the same. The distance between both transmitted beams can be controlled by adjusting the metafiber length and/or the rotation angle.

**
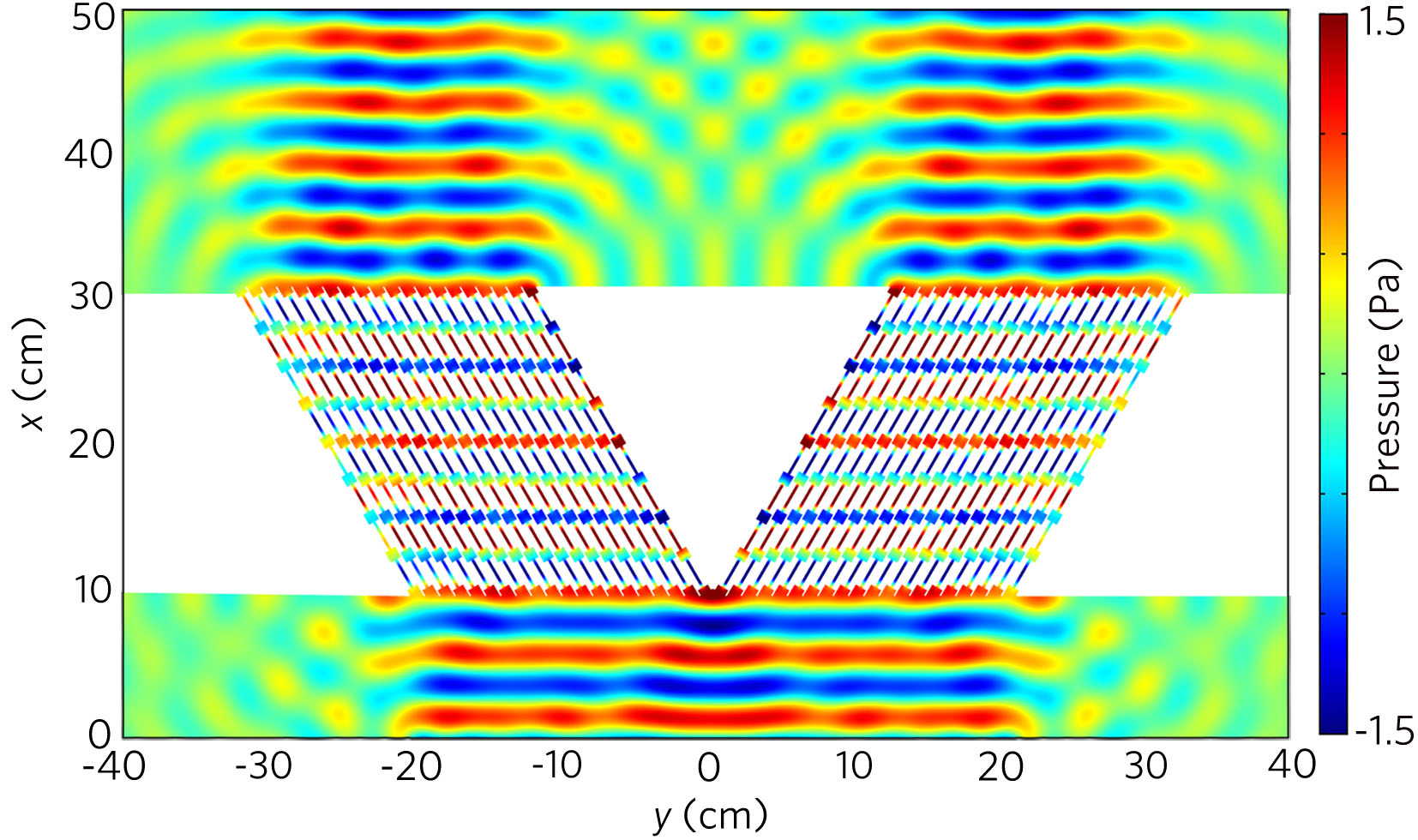
**

**Supplementary Figure 3 | Performances of acoustic beam splitter**. Simulation result of the acoustic pressure field distribution with the acoustic beam splitter illuminated by the normally incident plane wave at 8.0kHz.

**Supplementary Note 3**

**Broadband wavefront modulations**

The acoustic metafiber bundle has the capability of the wavefront modulations with broad bandwidth. Supplementary Figure 4 shows the distribution of the pressure field at 7.4kHz, 7.6kHz, 7.8kHz, 8.0kHz, 8.2kHz, 8.4kHz, 8.6kHz and 8.8kHz induced from the metafiber bundle (shown in Fig. 7b). It is clearly observed that the acoustic negative refraction exists at these frequencies, and all transmitted waves propagate along the designed direction (viz., 30^o^). The modulation bandwidth could reach about 1.4kHz, which further demonstrates the broadband performance of the metafiber bundle in the wavefront modulations.


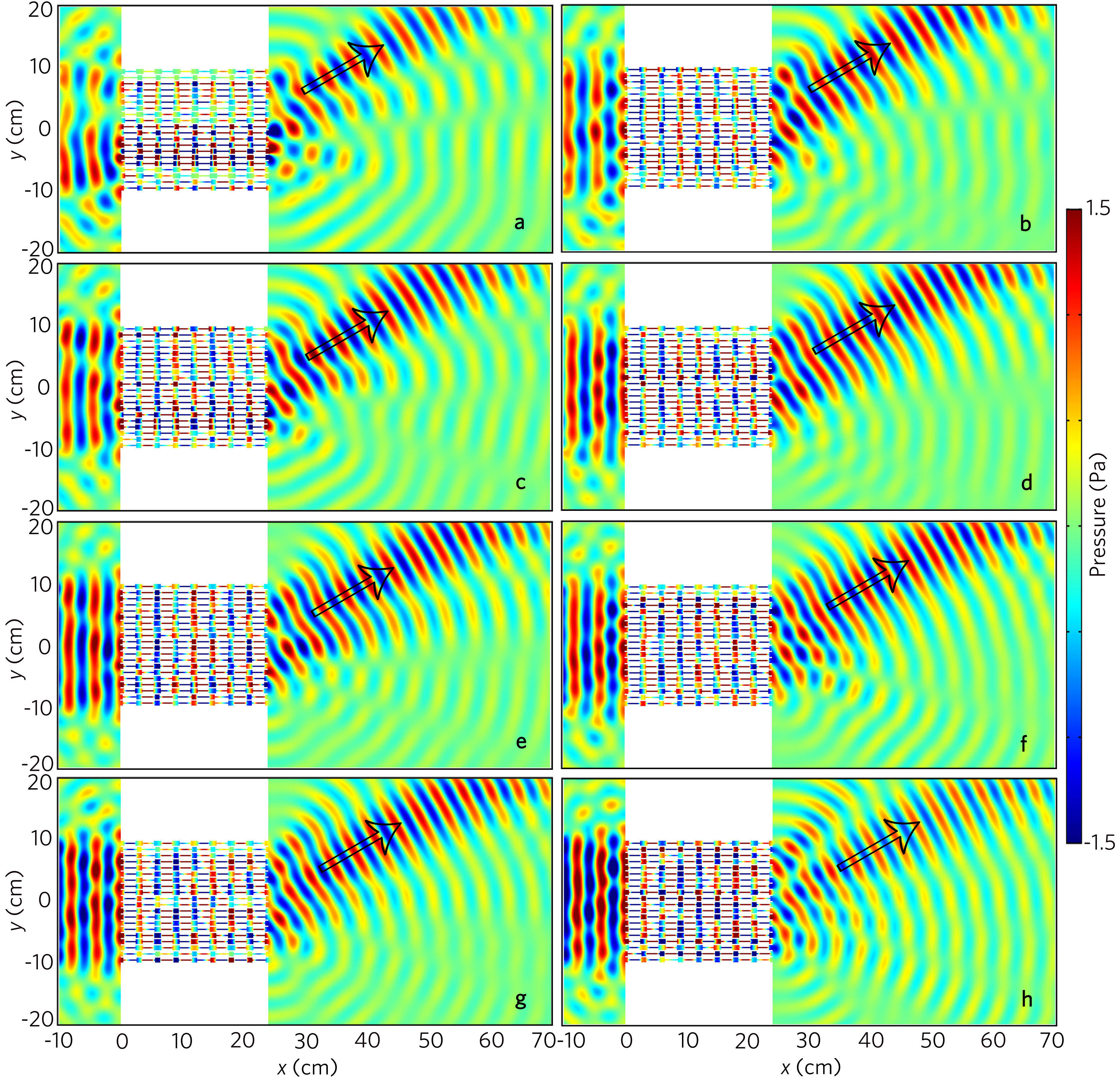


**Supplementary Figure 4 | Broadband acoustic metafiber bundle in wavefront modulations**. Simulation results of the acoustic pressure field distributions with the acoustic metafiber bundle illuminated by a normally incident plane wave with different frequencies. (**a**) 7.4kHz, (**b**) 7.6kHz, (**c**) 7.8kHz, (**d**) 8.0kHz, (**e**) 8.2kHz, (**f**) 8.4kHz, (**g**) 8.6kHz and (**h**) 8.8kHz. Black arrows in **a-h** refer to the theoretical values of the refraction.

**Supplementary Note 4**

**Complex wavefront modulations**

In this note, three examples of complex wavefront modulations for the cylindrical acoustic source are presented, such as the negative refractions with one or two beams and acoustic focusing.


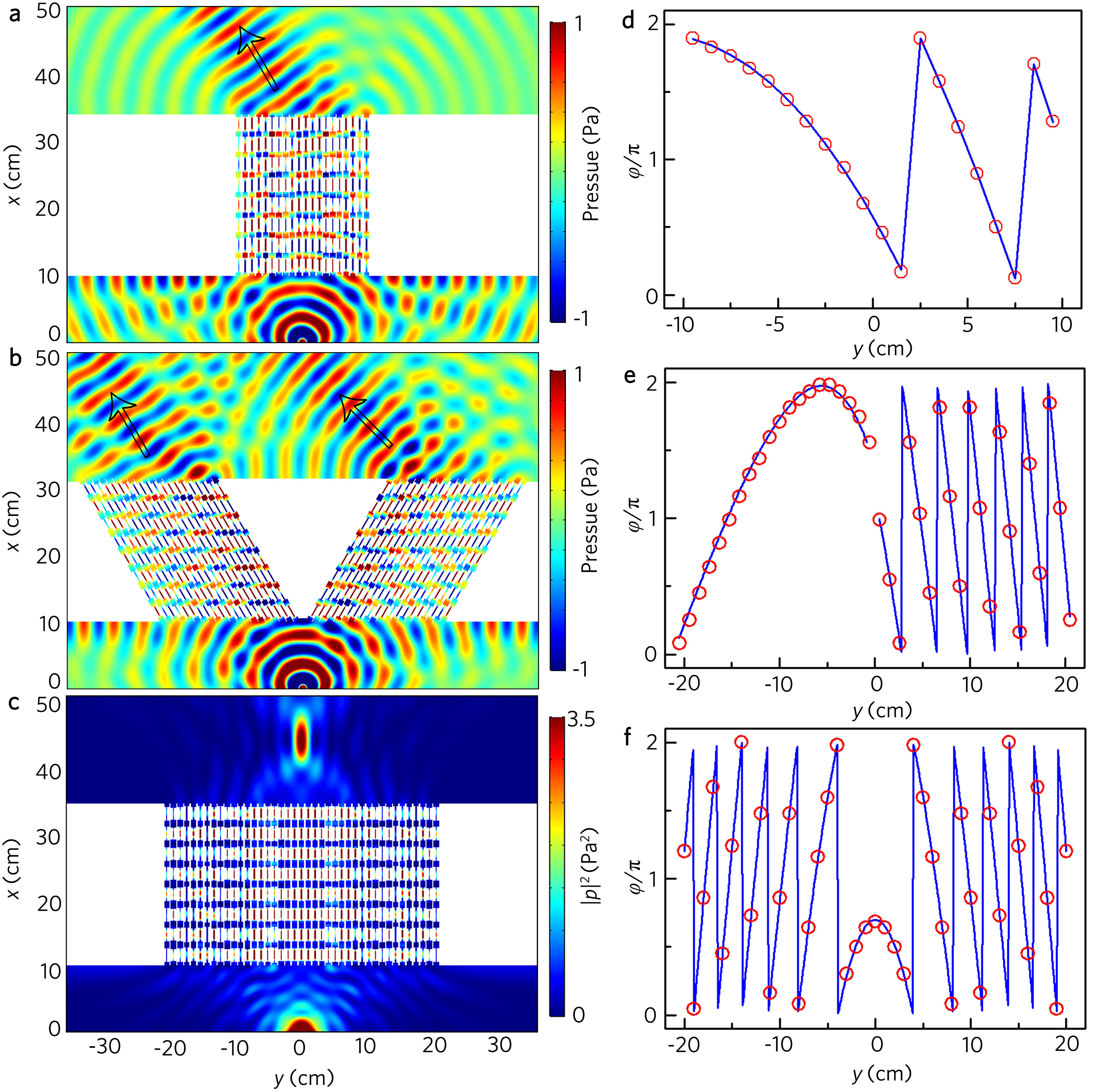


**Supplementary Figure 5 | Complex wavefront modulations with acoustic metafiber bundles for cylindrical acoustic wave.** Simulation results of the pressure field distributions for the negative refractions with (**a**) one or (**b**) two beams at 8.0 kHz. Simulation results of the intensity **|***p***|**^2^ field distribution for (**c**) acoustic focusing at 8.0 kHz. The cylindrical acoustic sources in **a**-**c** are located at (0, 0) in Cartesian coordinates, and the black arrows in **a** and **b** refer to the theoretical values of the refractions. The theoretical continuous phase shifts (blue lines) and the discrete phase shifts provided by the metafibers (red hollow points) along the *y* direction for the negative refractions with (**d**) one or (**e**) two beams and (**f**) acoustic focusing.

As shown in Supplementary Fig. 5a, we introduce an acoustic metafiber bundle composed of twenty metafibers. The cylindrical acoustic wave could transmit through the metafiber bundle, and is converted into the form of the plane acoustic wave along the designed propagation direction (viz., 30^o^). Supplementary Figure 5b shows the realization of the negative refractions with two beams for the cylindrical acoustic source, in which the metafiber bundle is composed of forty metafibers, and the distance between two metafibers and the rotation of the metafibers are the same as those in Fig. 8b. It shows that the cylindrical wave is converted into two acoustic beams with different propagation directions (viz., 30^o^ and 45^o^), which is different from Supplementary Fig. 3. The acoustic focusing for the cylindrical acoustic wave with *f*=10cm is shown in Supplementary Fig. 5c, in which the metafiber bundle is composed of forty-one metafibers. It shows that the cylindrical wave is focused as a perfect focal point. The aforementioned results indicate that the proposed metafiber bundles can be applied to the arbitrary complex waveform modulations for the cylindrical acoustic source. The corresponding distributions of three types of the acoustic bundles along the *y* direction are illustrated by the discrete phase shifts (shown by red hollow points) in Supplementary Fig. 5d-f, respectively, which is determined by the theoretical desired continuous phase profiles [cf. blue lines in Supplementary Fig. 5d-f]

**Supplementary References**

1. Li, Z. Y. & Lin, L. L. Photonic band structures solved by a plane-wave-based transfer-matrix method. *Phys. Rev. E*. **67**, 046607 (2003).
2. Fokin, V., Ambati, M., Sun, C. & Zhang, X. Method for retrieving effective properties of locally resonant acoustic metamaterials. *Phys*. *Rev*. *B* **76**, 144302 (2007).

1. ^1^ Research Center of Fluid Machinery Engineering and Technology, Faculty of Science, Jiangsu University, Zhenjiang 212013, China. ^2^ State Key Laboratory of Acoustics, Institute of Acoustics, Chinese Academy of Sciences, Beijing 100190, China. Correspondence and requests for materials should be addressed to H.-X.S. (email: jsdxshx@ujs.edu.cn) or to S.-Q.Y. (email: Shouqiy@ujs.edu.cn). [↑](#footnote-ref-2)
